# Evaluation of Osseointegration of Titanium Alloyed Implants Modified by Plasma Polymerization

**DOI:** 10.3390/ijms15022454

**Published:** 2014-02-11

**Authors:** Carolin Gabler, Carmen Zietz, Rebecca Göhler, Andreas Fritsche, Tobias Lindner, Maximilian Haenle, Birgit Finke, Jürgen Meichsner, Solvig Lenz, Bernhard Frerich, Frank Lüthen, J. Barbara Nebe, Rainer Bader

**Affiliations:** 1 Biomechanics and Implant Technology Research Laboratory, Department of Orthopaedics, University Medical Center Rostock, Doberaner Straße 142, 18057 Rostock, Germany; E-Mails: carmen.zietz@med.uni-rostock.de (C.Z.); rebecca.goehler@uni-rostock.de (R.G.); a.fritsche@deru-hamburg.de (A.F.); tobias.lindner@med.uni-rostock.de (T.L.); mjm@haenle.org (M.H.); rainer.bader@med.uni-rostock.de (R.B.); 2 Leibniz Institute for Plasma Science and Technology (INP e.V.) Greifswald, Felix-Hausdorff-Str. 2, 17489 Greifswald, Germany; E-Mail: finke@inp-greifswald.de; 3 Institute of Physics, University of Greifswald, Felix-Hausdorff-Str. 6, 17487 Greifswald, Germany; E-Mail: meichsner@physik.uni-greifswald.de; 4 Department of Oral and Maxillofacial Surgery, University Medical Center Rostock, Schillingallee 35, 18057 Rostock, Germany; E-Mails: solvig.lenz@uni-rostock.de (S.L.); bernhard.frerich@med.uni-rostock.de (B.F.); 5 Department of Cell Biology, University Medical Center Rostock, Schillingallee 69, 18057 Rostock, Germany; E-Mails: frank.luethen@med.uni-rostock.de (F.L.); barbara.nebe@med.uni-rostock.de (J.B.N.)

**Keywords:** implant coating, plasma polymerization, cell adhesion, animal experiment, osseointegration

## Abstract

By means of plasma polymerization, positively charged, nanometre-thin coatings can be applied to implant surfaces. The aim of the present study was to quantify the adhesion of human bone cells *in vitro* and to evaluate the bone ongrowth *in vivo*, on titanium surfaces modified by plasma polymer coatings. Different implant surface configurations were examined: titanium alloy (Ti6Al4V) coated with plasma-polymerized allylamine (PPAAm) and plasma-polymerized ethylenediamine (PPEDA) *versus* uncoated. Shear stress on human osteoblast-like MG-63 cells was investigated *in vitro* using a spinning disc device. Furthermore, bone-to-implant contact (BIC) was evaluated *in vivo*. Custom-made conical titanium implants were inserted at the medial tibia of female Sprague-Dawley rats. After a follow-up of six weeks, the BIC was determined by means of histomorphometry. The quantification of cell adhesion showed a significantly higher shear stress for MG-63 cells on PPAAm and PPEDA compared to uncoated Ti6Al4V. Uncoated titanium alloyed implants showed the lowest BIC (40.4%). Implants with PPAAm coating revealed a clear but not significant increase of the BIC (58.5%) and implants with PPEDA a significantly increased BIC (63.7%). In conclusion, plasma polymer coatings demonstrate enhanced cell adhesion and bone ongrowth compared to uncoated titanium surfaces.

## 1. Introduction

Total joint replacement (TJR) has become a routine surgical procedure in order to restore joint function. TJR is associated with high quality and safety standards [1]. Implant revision is, however, a crucial problem in clinical use. Most failures of TJR are caused by aseptic loosening due to the inflammatory reactions as a result of the wear particles [2,3]. Implant-associated infections occur only with a low rate of 0.5% to 5% but are related to severe complications for the patient and high costs for the healthcare system [3,4].

For long-term implant survival, an appropriate bone ongrowth without fibrous tissue interface is required [5]. After insertion of the implant, the so-called “race for the surface” describes the competition between human bone cells and bacteria for its surface in the early postoperative period [6]. The adherence of bacteria to the implant surface prior to human bone cells leads to biofilm formation and can impede osseointegration of the implant.

The adhesion and ongrowth of bone cells can be supported by an adequate functionalization of the implant surface [7–9]. By means of plasma polymerization, positively charged, nanometre-thin coatings can be applied to the implant surfaces [10]. In particular, nitrogen-rich plasma-polymer coatings by use of the monomers allylamine and ethylenediamine with nitrogen functional groups, seem to be suitable for the enhancement of adhesion and ongrowth of human osteoblasts [11]. *In vitro* investigations confirm the advanced ongrowth of bone cells on plasma-polymerized allylamine (PPAAm)- or ethylenediamine (PPEDA)-coated titanium surfaces [12,13]. Animal studies investigating the immunological response of these surface modifications showed an immunological reaction comparable to untreated surfaces [14–16].

The aim of the present study was to analyse and quantify the adhesion behaviour of human bone cells on plasma-modified PPAAm and PPEDA implant surfaces *in vitro*, as well as to evaluate the bone ongrowth using these plasma-modified implant surfaces by means of animal testing in rats.

## 2. Results and Discussion

### 2.1. Cell Adhesion

**Figure 1.**
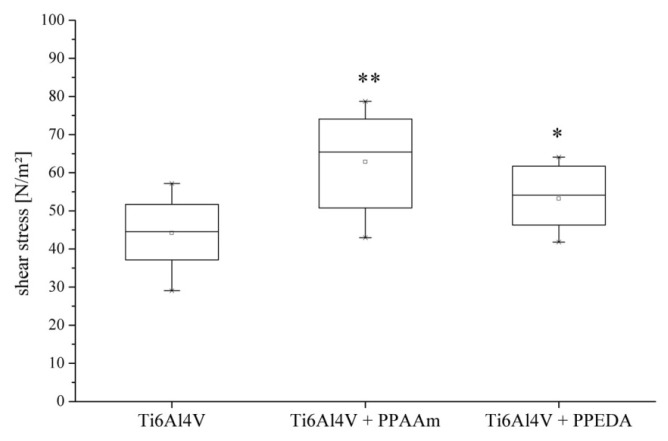
Shear stress (N/m^2^) exerted on MG-63 cells deployed on different implant surface configurations (uncoated, PPAAm-coated and PPEDA-coated titanium discs). Shear stress on plasma polymer coatings was significantly higher compared to uncoated Ti6Al4V (* *p* ≤ 0.05; ** *p* ≤ 0.001).

The adhesion test data demonstrated that bone cell (MG-63) adherence was lowest on uncoated Ti6Al4V (44.2 ± 9.0 N/m^2^) discs and increased on PPAAm-coated (66.8 ± 12.0 N/m^2^) and PPEDA-coated (53.2 ± 8.3 N/m^2^) surfaces. After 18 h incubation time, the MG-63 cells revealed significantly higher shear stress on PPAAm- (*p* ≤ 0.001) and PPEDA-coated samples (*p* = 0.03) compared to uncoated samples indicating a highly cell-attractive polymer layer. Between plasma coatings, significant differences (*p* = 0.31) were not found (Figure 1).

### 2.2. Bone-to-Implant Contact

**Figure 2.**
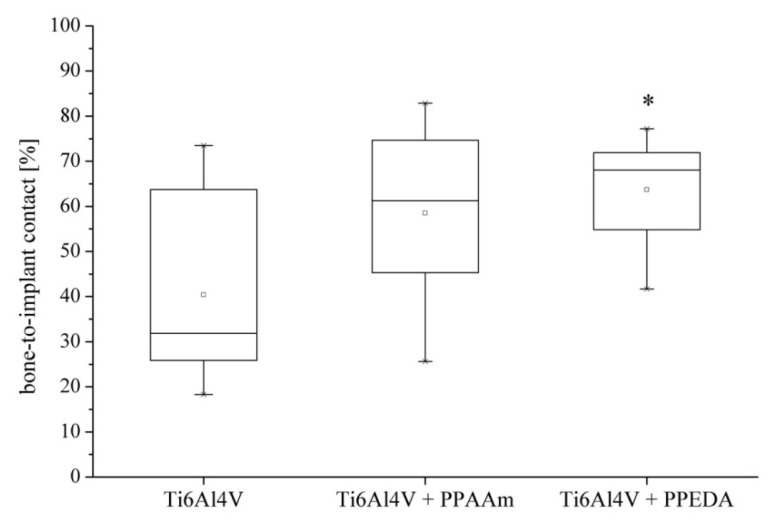
Bone-to-implant contact (BIC) (%) of the implants with different titanium implant surface configurations (uncoated, PPAAm-coated and PPEDA-coated) after six weeks *in vivo*. BIC was increased by plasma polymer coatings (* *p* ≤ 0.05).

During the six-week implantation period, no signs of implant-associated infection or other alterations in the animal behaviour were observed. All plasma-coated and uncoated titanium implants showed direct bone contact in histological analysis. However, there were differences in the extent of bone-to-implant contact (BIC) among the types of coating. The uncoated titanium implants showed the lowest BIC with a mean value of 40.4% (standard deviation (SD) = 20.8%) after six weeks of implantation. The plasma-polymerized coatings resulted in a considerable increase of the BIC. The PPAAm coating demonstrated a mean value of 58.5% (SD = 22.2%); however, the increase of the PPAAm-coated implants were not significant compared to uncoated implants (*p* = 0.16). The mean value for the PPEDA coating (63.7%, SD = 11.9%) was significantly higher (*p* = 0.03). Between PPAAm and PPEDA, there was no significant difference (*p* = 0.60; Figure 2).

### 2.3. Discussion

Due to its interactions with bone cells and bacteria, the surface characteristics of orthopaedic implants play an important role in the success or failure of implants for total joint replacement. Osteoblast functions can be promoted by influencing the surface topography (e.g., rough surfaces) on the one hand [7,8] and, on the other hand, the cellular adhesion can be influenced by changing the surface chemistry [7,9]. For example, calcium phosphate (CaP) coatings are known to increase the saturation of body fluids whereby a biological apatite layers on the implant surface [17,18]. Several studies have shown promise concerning enhanced bone contact and growth [19–22]. Dental implants coated with plasma-sprayed CaP showed better clinical success [23] as well as the same long-term implant survival compared to other surface types [24]. One of the major problems is the possible delamination of the CaP coating from the implant surface, which could result in clinical problems [24,25]. Functionalization of surfaces with RGD peptides was shown to enhance osteoblast function but not bacterial adhesion [26–28]. Even positive *in vivo* results have been observed [29–32]. Thereby, a lot of basic work is aimed at finding sequences specifically targeted to bone cells and to overcome the “universality” and lack of selectivity of the RGD-signal [33]. Attention was directed towards bone morphogenetic proteins (BMPs). This class of signalling molecules is known to promote osseoconductive and osseoinductive bone formation [34,35]. Shi *et al*. tried to achieve the dual purpose of increased osteoblast function and inhibited bacterial colonization on titanium alloy surfaces [36,37]. Therefore they immobilized BMP-2 with either an anti-adhesive polymer or bactericidal polymer as an intermediate layer. The dextran layer inhibited the adhesion of both bacteria and osteoblasts. With BMP-2 grafted onto the dextran surface with a surface protein density of >50 ng/cm^2^, osteoblast spreading was promoted without reducing the antibacterial efficacy. Nevertheless, after 6 h culture, the number of attached osteoblasts was only ~80% of that on the uncoated titanium surface. After 24 h cultivation, the cell coverage increased compared to that on untreated titanium [37].

In the present experimental study, new plasma-modified titanium implant surfaces were analysed *in vitro* and *in vivo* in comparison to uncoated surfaces. The test results demonstrated positive effects of plasma-polymerized allylamine (PPAAm) and ethylenediamine (PPEDA) on cell adhesion and bone ongrowth. Both plasma coatings showed a significant increase in shear stress of MG-63 cells, which were seeded on the coated test samples and incubated for 18 h. The quantitative cell adhesion investigations confirmed the data of prior qualitative cell adhesion and cell spreading tests [12,13]. In the *in vivo* investigations, we could demonstrate a higher bone-to-implant contact (BIC) for both plasma coatings compared to uncoated titanium implants after six weeks of implantation. The BIC for PPEDA-coated implants was significantly higher compared to uncoated implants. Although, if the increase of BIC for PPAAm-coated implants was not significant, a clear positive trend for enhanced bone ongrowth was found.

The investigations *in vivo* and *vitro* required different surface treatments. The *in vitro* investigations of the adhesive strength had to be performed at polished surfaces (*R*z = 0.13 μm) in order to ensure laminar flow conditions during spinning the test samples. For *in vivo* investigations of bone ongrowth we used rough samples (*R*z = 20 μm) with the same surface characteristics of standard titanium implants provided for cementless bone fixation.

The response of human osteoblasts to the plasma-polymerized surfaces was only carried out without consideration of bacterial colonization so far. Studies are planned to investigate how bacteria adhere to the plasma-polymerized coatings. It should be demonstrated that bacteria compared to human osteoblast show no increased affinity to PPAAm- or PPEDA-coated surfaces. Hence, a positive influence on the “race for the surface” [6] of bone cells against bacteria could have beneficial effects on the osseointegration of plasma-polymerized titanium implants in future clinical application.

The present animal study was limited by a low number of samples for the histomorphometrical evaluation. We could only examine 20 implants and only one slice per implant was made to determine the BIC. Nevertheless, *in vivo* data of BIC confirms the *in vitro* results of the shear stress test. Before the histological preparation, the rats’ tibiae with implants were therefore scanned via micro-computed tomography (μ-CT). The evaluation of the μ-CT data is still in progress in order to compare the data of the overall implant surface with the results from a two-dimensional slice of the implants analysed by means of histomorphometry.

## 3. Experimental Section

### 3.1. Implant Material and Surface Coatings

For the *in vitro* tests, disc-shaped samples and, for the *in vivo* tests, custom-made implants were used. All specimens were made of titanium alloy (Ti6Al4V). Three different implant surface configurations were examined: uncoated, plasma-polymerized allylamine (PPAAm) and plasma-polymerized ethylenediamine (PPEDA).

The PPAAm coating was applied in a two-step procedure. First, the titanium sample was decontaminated and activated by continuous-wave oxygen plasma (500 W, 50 Pa, 100 sccm O_2_/25 sccm Ar). Then the monomer allylamine was plasma-polymerized by the use of a pulsed (duty cycle of 0.15 at a pulse length of 2 s) microwave plasma source (2.45 GHz, 500 W) under low pressure (50 Pa) for an effective treatment duration of 144 s using argon-allylamine gas mixture [10].

For the PPEDA coating, a capacitively coupled radio frequency discharge (13.56 MHz) was used as plasma source. The metallic implant surface acted as electrode for the discharge. The implant coating was applied by use of an argon-ethylenediamine gas mixture, a total pressure of 60 Pa and radio frequency forward power between 60 and 100 W [10,38].

The thickness of tested PPAAm and PPEDA coatings were approximately 50 nm [10]. The adhesive bonding strength of the plasma polymerised coatings were according to test standard DIN EN 582 [39]. Coated cylindrical test samples made of titanium with 25 mm diameter were used. The adhesive bonding strengths of the coatings were higher than the 22 N/mm^2^ required for medical implant surface coatings by ASTM standard 4711-F [40].

### 3.2. Cell Adhesion

The quantification of cell adhesion was established by Fritsche *et al*. using a spinning disc device [41]. The disc-shaped Ti6Al4V samples (diameter: 30 mm, height: 8 mm), manufactured by DOT GmbH (Rostock, Germany), with polished surfaces were uncoated (*n* = 20, *R*z = 0.13 μm) as well as coated with PPAAm (*n* = 22, *R*z = 0.16 μm) and PPEDA (*n* = 10, *R*z = 0.18 μm). Across the diameter of the discs, several positions were marked by a laser to determine the radial distance of cell detachment. The radial distance of the marked positions can be used to calculate the shear stress.

Before cell seeding, all test samples underwent a cleaning process including ultrasonic bath, rinsing with tap water and distilled water and blow-drying. Sterilization was performed by covering the samples with 70% ethanol and air-drying in a laminar flow box.

The fluorescent dye PKH26 (Sigma-Aldrich, St. Louis, MO, USA) was used to stain the membrane of vital bone cells [12,13]. Approximately 2000 MG-63 cells were then seeded in 2 μL droplets of Dulbecc’s Modified Eagle’s Medium (DMEM) with 10% foetal calf serum (FCS, Gibco Invitrogen, Karlsruhe, Germany) and 1% gentamicin (Ratiopharm, Ulm, Germany). The droplets were applied onto the discs in a row and subsequently joined as a fluid stripe across the radial laser-marked positions. The discs were incubated for 20 min at 37 °C and 5% CO_2_. Subsequently, the polished discs were covered with 4 mL DMEM and incubated again until a total incubation time of 18 h.

The discs were mounted into the test device. The test chamber was filled with 37 °C tempered DMEM. Microscopic images of each laser-marked position were taken by a confocal laser scanning microscope (LSM 410, Zeiss, Oberkochen, Germany) before spinning. A spinning time of 3 min was used. After spinning-off, the positions were scanned again. For each position, the microscopic images before and after rotation were analysed and the cells were counted using a custom-made image analysing tool, programmed with Python 2.6 (Python Software Foundation, Wolfeboro Falls, NH, USA).

The MG-63 cells were subject to medium-mediated shear stress during the rotation of the disc, which causes their detachment. The magnitude of the shear stress depends on the angular velocity and the radial position of the cells on the disc. In addition, the viscosity and density of the medium must be considered. Considering all shear stress components and ideal laminar flow conditions, the resulting shear stress can be calculated [41,42].

### 3.3. Animal Testing

**Figure 3.**
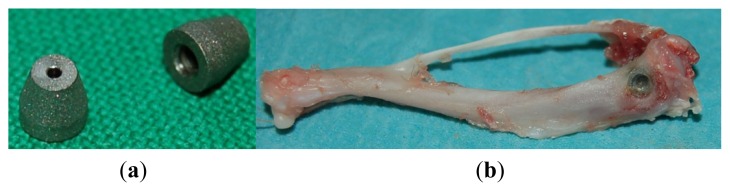
(**a**) Implant for insertion in the tibial metaphysis of the Sprague-Dawley rats; (**b**) Retrieved tibial bone with an implant in the medial metaphysis.

The animal experiments were approved by the local review board of the Landesamt für Landwirtschaft, Lebensmittelsicherheit und Fischerei M-V (LALLF MV, Reference number 7221.3-1.1-031/09). A custom-made conical Ti6Al4V implant (3 mm maximum outer diameter and 3 mm length) [43], manufactured by primec GmbH (Bentwisch, Germany), with a rough surface (blasted by hydroxyapatite, *R*z = 20 μm), was used (Figure 3a). The implant surfaces investigated were uncoated as well as coated with PPAAm and PPEDA.

Surgery was performed on female Sprague-Dawley rats (weight: 289 ± 22 g) under general anaesthesia induced by an intramuscular injection of 150 μg/kg medetomidine (Dorbene vet^®^, Fort Dodge, Würselen, Germany), 200 μg/kg midazolam (Ratiopharm, Ulm, Germany) and 5 μg/kg fentanyl (Ratiopharm). The skin of the hind limbs was shaved and disinfected before sterile draping. The medial aspect of the tibial metaphysis was exposed through a medial incision of skin and fascia. Using a circular drill (2.8 mm in diameter), a bone defect was prepared bilaterally at the medial proximal tibiae. The implants were inserted into the defects (Figure 3b). Surgical sites were closed using sutures (Vicryl^®^ 5-0, Ethicon, Somerville, NJ, USA). Subsequently, anaesthesia was antagonized by a subcutaneous injection of 750 μg/kg atipamezol (Alzane^®^, Pfizer, Berlin, Germany), 200 μg/kg flumazenil (Ratiopharm, Ulm, Germany) and 120 μg/kg naloxon (Ratiopharm, Ulm, Germany).

A total of 16 rats (for experiments with uncoated implants: 6; for PPAAm: 5; for PPEDA: 5) were observed for six weeks. The animals were sacrificed and subsequently the tibiae were carefully dissected and freed of soft tissue (Figure 3b) and fixed in buffered formalin (4%) until histomorphometric processing. A total of 20 implants were used for histomorphometric analysis. The remaining implants were scanned via micro-computed tomography (μ-CT) or were used for microbiological investigations [43].

### 3.4. Histomorphometric Analysis

**Figure 4.**
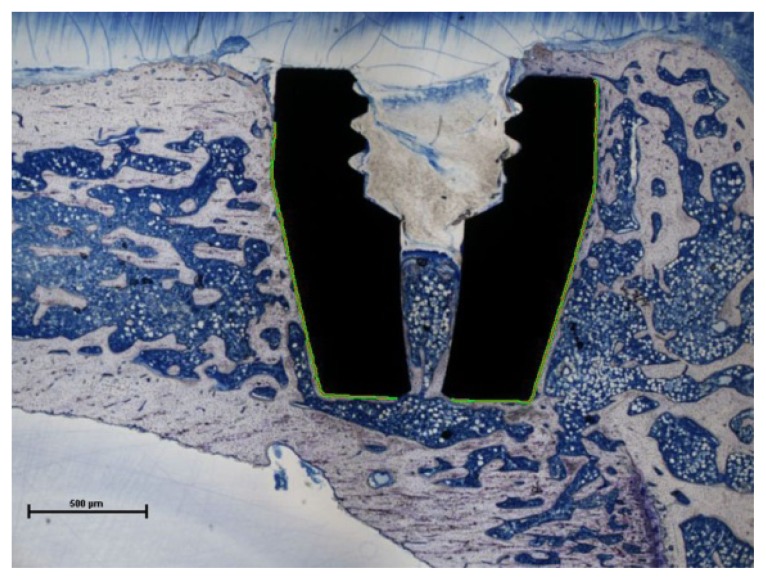
Histomorphometric sample with a PPAAm-coated implant in the proximal rat tibia (toluidine blue staining). Green line marks the evaluated implant region for BIC.

The specimens (uncoated: 7; PPAAm: 6; PPEDA: 7) were dehydrated in a graded series of alcohol and embedded in polymethylmetacrylate. For each implant, one slice in the longitudinal direction of the implant was ground and stained with toluidine blue (Figure 4).

Histological evaluations were performed using a light microscope (Eclipse TS 100; Nikon, Japan) equipped with a camera (Nikon Digitale Sight DS-2 mV) and connected to a computer. The histomorphometric data were analysed by software for microscopic images, NIS-Elements D 3.2 (Nikon Instruments Inc., Melville, NY, USA).

Only those parts of the implant were considered, that were completely surrounded by bone tissue. Bone-to-implant contact (BIC) was determined as the percentage from the surface length overgrown with bone tissue to the total surface contact length of the implant.

### 3.5. Statistical Analysis

The statistical analysis was performed using SPSS Statistics 20 (IBM, Armonk, NY, USA). The statistical data includes mean and standard deviation. Pair-wise comparisons within the independent groups were performed using the *t*-test. All values were proven to have normal distribution by the Shapiro-Wilk test and homogeneity of variance before the *t*-test. The *p*-values < 0.05 were considered as statistically significant.

## 4. Conclusions

Titanium alloyed surfaces modified by plasma polymerization, *i.e.*, coating by PPAAm and PPEDA, showed increased adhesive strength of human MG-63 osteoblastic cells *in vitro*. Furthermore, the plasma-polymerized coatings enhanced the bone-to-implant contact compared to uncoated Ti6Al4V implants *in vivo*. These results promote PPAAm and PPEDA as clinically relevant coatings that can provide positive influence on the “race for the surface” of bone cells, which may have beneficial effects on the osseointegration of titanium implants in future clinical applications.
